# Anatomic Posterolateral Corner Reconstruction of the Knee Using Single Semitendinosus Autograft: Technical Note for the Borderline-length Graft

**DOI:** 10.1055/s-0044-1793826

**Published:** 2024-12-21

**Authors:** Francisco Lima-Bernardes, Nuno Vieira da Silva, Pedro Ribeiro, Diogo Manuel Soares, Nuno Ferreira, Hélder Nogueira

**Affiliations:** 1 Serviço de Ortopedia, Centro Hospitalar do Tâmega e Sousa, Penafiel, Portugal

**Keywords:** knee, igaments, articular, tendon injuries

## Abstract

The original LaPrade technique for anatomic reconstruction of the posterolateral corner of the knee uses two separate allografts. More recently, a modification of this technique, using an adjustable-length suspension device with a cortical button for tibial fixation, allows anatomic reconstruction with a single semitendinosus autograft. This modification is of utmost relevance when sources of allograft are not available for multiligament knee reconstruction. In both techniques interference screws are used for femoral fixation of the fibular collateral ligament and popliteus tendon. The minimum length recommended for the anatomic reconstruction with single semitendinosus is 25 cm, but anatomic variations in the population exist, and a longer semitendinosus may be necessary. Indeed, some patients may only reach the necessary length considering the thinnest limb of the semitendinosus. In these patients, femoral fixation of the thinnest limb with a knotless suture anchor, as we describe, for the popliteus tendon limb, allows expansion of this technique to borderline semitendinosus autografts while reducing the risk of tunnel coalition. We also describe a different sequence of steps: fixation of the fibular collateral ligament in the femoral tunnel followed by its tensioning and fixation in the fibular head tunnel, fixation of the popliteus tendon in its femoral footprint with a knotless suture anchor and, finally, tensioning of the popliteofibular ligament and popliteus tendon. This different sequence also helps avoiding tendon waste, which may be left over, allowing more graft incorporation into the tibial tunnel.

## Introduction

The posterolateral corner (PLC) of the knee consists of three major static stabilizers which restrain varus and external rotation: the fibular collateral ligament (FCL), the popliteus tendon (PT), and the popliteofibular ligament (PFL). The mechanism of injury involves varus stress, hyperextension and twisting of the knee. Posterolateral corner insufficiency may result in meniscal injuries and accelerated medial compartment osteoarthritis. 1 Posterolateral corner injuries rarely occur in isolation, being typically associated with injury to one or both cruciate ligaments. In the setting of a multiligament injury, failure to address concomitant PLC insufficiency leads to increased forces on the reconstructed cruciate ligaments and may lead to surgical failure. 2 3

Many techniques, both anatomic and nonanatomic, have been described. Although some studies present similar clinical outcomes of anatomic and nonanatomic techniques, 4 some biomechanical studies favor anatomic PLC reconstruction. 1 3 5 The original anatomic technique, published by LaPrade et al. 1 requires two separate allografts of the Achilles tendon: one for the FCL and PFL and another for the PT. Modifications of the technique, in which two separate hamstrings autografts are used, have also been described. 3 6 However, since PLC lesions commonly occur in association with other ligament injuries, harvesting two hamstrings for the PLC reconstruction increases graft harvesting morbidity. In 2019, Wood et al *.* 2 described a modification of the LaPrade et al. 1 technique using an adjustable-length suspension device with a cortical button for tibial fixation of the PFL and PT limbs, allowing PLC reconstruction with a single semitendinosus (ST) autograft. This technique modification is a game changer when sources of allograft are not available for multiligament knee reconstruction. The minimum ST length recommended by the authors is 25 cm. 2 However, there are anatomic variations in the population 7 8 ( Table 1 ), and a longer ST may be necessary. In our experience, some patients may only reach the necessary length considering the thinnest limb of the ST.

**Table 1:** Anatomic considerations for graft length estimation 1 7 8

Anatomic structure	Graft length needed in millimeters (range)
Fibular collateral ligament	69.6 (62.6–73.5) 7
Popliteus tendon	54.5 (50.5–61.2) 7
Popliteofibular ligament	14.7 (12.2–17.2) 8
Fibular head tunnel	36.3 (31.2–40.7) 7
Femoral tunnels	50 1 2
Tibial tunnel	Remaining graft length

**Fig. 1:**
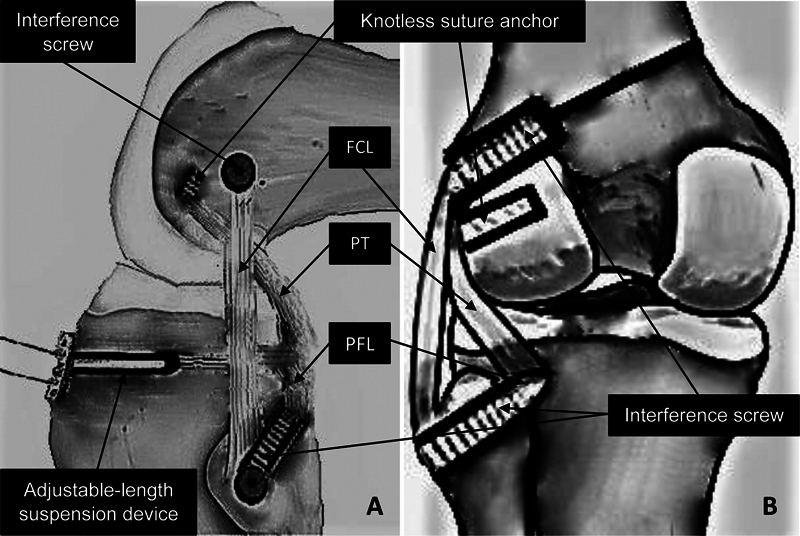
Left knee, schematic representation of the surgical technique. Lateral view ( **A** ) and posterior view ( **B** ). **Abbreviations:** FCL, fibular collateral ligament; PFL, popliteofibular ligament; PT, popliteus tendon.

The objective of this technical note is to describe a modification of the Wood et al *.* 2 technique with femoral fixation of the thinnest limb of the graft with a knotless suture anchor ( Fig. 1 ). We also propose a different sequence of steps to help reduce tendon waste and allow more incorporation of the graft into the tibial tunnel.

## Surgical Technique

The last author was the leading surgeon and the one who developed the technique.

The patient was positioned supine, with a lateral thigh post and a footrest at 90° flexion. A tourniquet was placed around the proximal thigh and inflated as required.

As described by LaPrade et al. 1 and Wood et al., 2 a lateral approach including neurolysis of the common peroneal nerve was performed. Then, a horizontal incision was made over the tendon of the long head of the biceps femoris with exposure of the fibular head and identification of the FCL remnant, which was tagged with traction sutures, allowing the identification of its femoral and fibular footprints. Subperiosteal dissection of the lateral aspect of the fibular head was then performed with exposure of the “champagne glass drop-off transition” and the fibular head tunnel was created (6 × 35 mm), from anterolateral to posteromedial, with the aid of a proper fibular head aiming device (Arthrex Inc., Naples, FL, USA). Then, distally and medial to the Gerdy's tubercle, the “flat spot” on the anterior tibia was identified as well as the musculotendinous junction of the popliteus on the posterior tibia, proximally and medially do the fibular head tunnel. A proper tibial aiming device (Arthrex Inc.) was used, and a complete tibial tunnel for the PFL and PT was created. A second horizontal incision was made on the iliotibial band at the level of the lateral epicondyle with identification of the femoral footprint of the FCL, and an L-shaped capsulotomy was made allowing visualization of the PT footprint on the anterior popliteal sulcus. 1 2

**Fig. 2:**
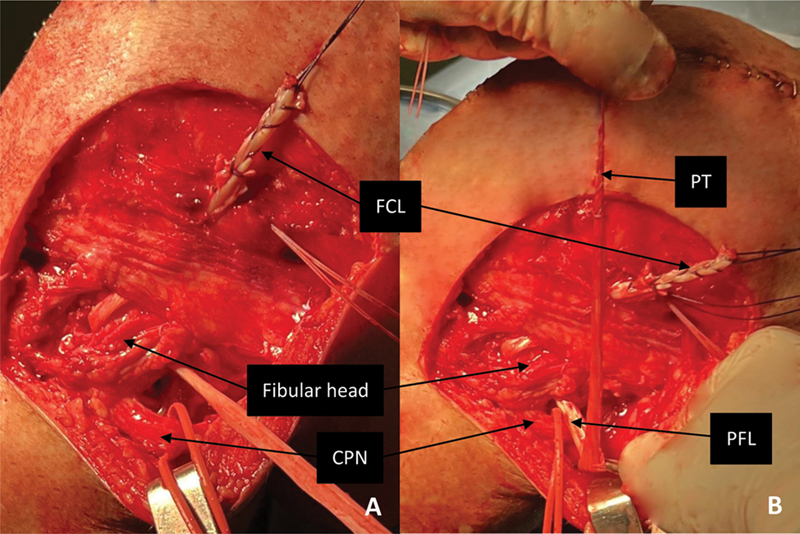
Left knee, lateral view. Passage of the graft into the fibular head tunnel ( **A** ) and initial estimation of the necessary semitendinosus length for single graft reconstruction ( **B** ). **Abbreviations:** CPN, common peroneal nerve; FCL, fibular collateral ligament; PFL, popliteofibular ligament; PT, popliteus tendon.

**Fig. 3:**
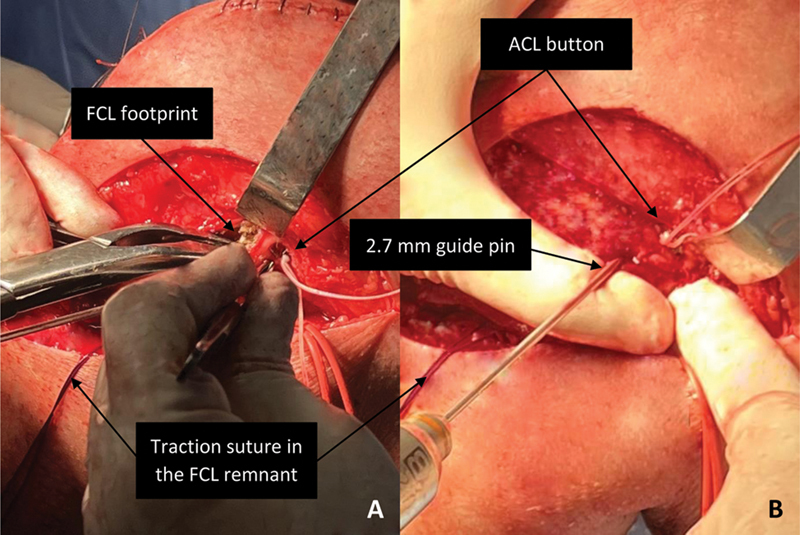
Left knee, lateral view. Preparation of the FCL footprint ( **A** ) and guide pin placement prior to tunnel drilling ( **B** ). **Abbreviations:** ACL, anterior cruciate ligament; FCL, fibular collateral ligament.

**Fig. 4:**
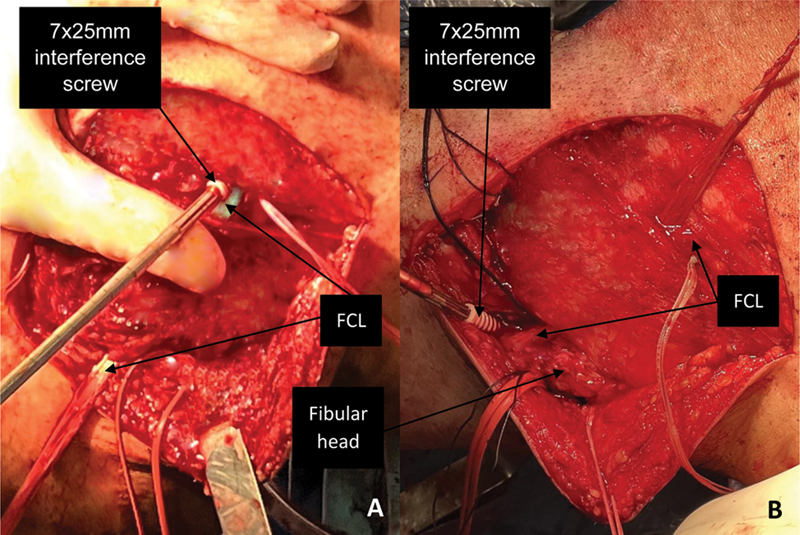
Left knee, lateral view. Fixation of the FCL in the femoral tunnel ( **A** ) with a 7 × 25-mm interference screw (BioComposite FastThread, Arthrex Inc.). Fixation of the FCL in the fibular head tunnel ( **B** ) with a 7 × 25-mm screw (BioComposite FastThread, Arthrex Inc.) in neutral rotation, 30° flexion and valgus stress. **Abbreviation:** FCL, fibular collateral ligament.

**Fig. 5:**
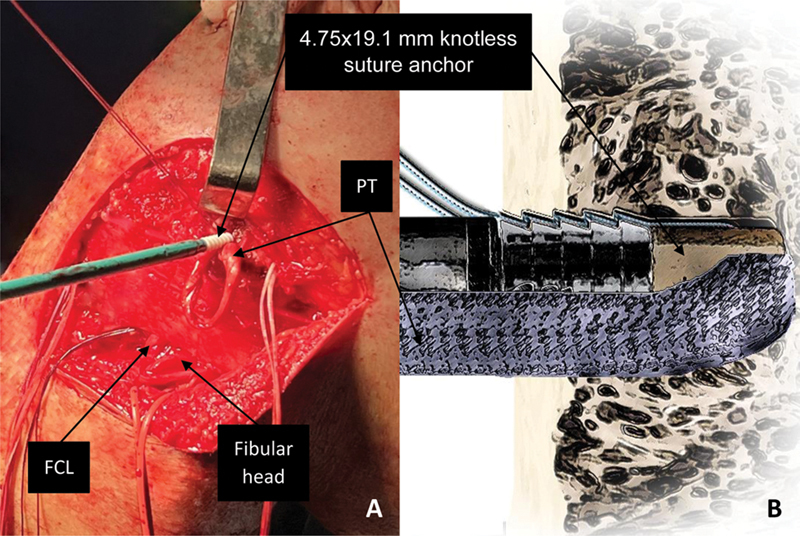
Left knee, lateral view. Fixation of the PT in its femoral footprint ( **A** ) with a 4.75 × 19.1-mm knotless suture anchor (BioComposite SwiveLock, Arthrex Inc.). Knotless suture anchor detail with tendon interposition ( **B** ). **Abbreviations:** FCL, fibular collateral ligament; PT, popliteus tendon.

**Fig. 6:**
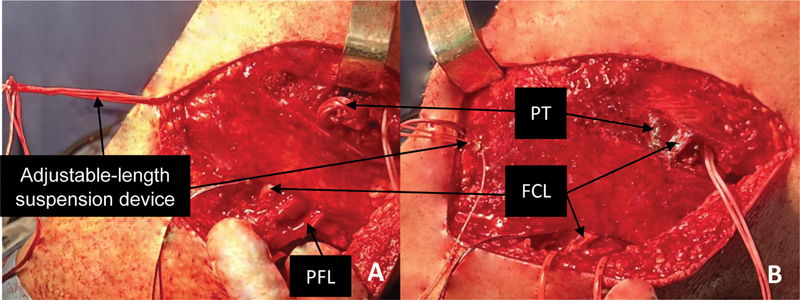
Left knee, lateral view. PT and PFL prior to tensioning ( **A** ) and after tensioning ( **B** ). Tensioning is made in 60° flexion and neutral rotation with a cortical button mounted on an adjustable-length suspension device (Attachable Button System, Arthrex Inc.). **Abbreviations:** FCL, fibular collateral ligament; PFL, popliteofibular ligament; PT, popliteus tendon.

The harvested ST measuring 27 cm, considering its thinnest extremity of 2.5 cm, was tubularized with whipstitched sutures for easy passage into the tunnels of the femur, fibular head, and tibia. The graft was passed into the fibular head tunnel ( Fig. 2A ), and an initial estimation of the necessary length was made ( Fig. 2B ). For femoral fixation of the FCL, after preparation of the FCL footprint ( Fig. 3A ) and proper guide pin placement ( Fig. 3B ), a 6 × 25-mm tunnel was created, and a passing suture was left in place. Sequential fixation of the graft then took place. First, the thickest extremity of the graft was pulled into the femoral tunnel and secured with a 7 × 25-mm interference screw (BioComposite FastThread - Arthrex Inc., Fig. 4A ). Then, the graft was fixed in the fibular head with a 7 × 25-mm screw (BioCompsite FastThread - Arthrex Inc, Fig. 4B ) in neutral rotation, 30° flexion and valgus stress. The graft was then passed through the loop of an adjustable-length suspension device (Attachable Button System- Arthrex Inc.). The suspension device was then passed anteriorly through the tibial tunnel, and a cortical button was applied. The PT limb was then passed along the popliteal hiatus to its footprint. Since this extremity of the graft was not thick enough for interference screw fixation, a knotless suture anchor was used instead. In more detail, the popliteus tendon limb was whipstitched and passed through the eyelet of a 4.75 × 19.1-mm knotless suture anchor which was applied in its footprint (BioComposite SwiveLock - Arthrex Inc., Figs. 5A,B ). The PT and PFL were then tensioned in 60° flexion and neutral rotation ( Figs. 6A,B ). After fixation of all structures, knee stability and range of motion were tested. Following copious irrigation of the tissues, the lateral capsule, iliotibial band, subcutaneous tissue, and skin were closed in a standard fashion. The main surgical steps are summarized in Table 2 .

**Table 2:** Summarized surgical steps

Surgical step	Details
Positioning	Supine with a lateral thigh post and a footrest at 90° flexion.
Approach	Lateral approach as described by LaPrade et al *.* 1 and Wood et al *.* 2 including neurolysis of the common peroneal nerve.
Fibular head tunnel preparation	Subperiosteal dissection of the lateral aspect of the fibular head exposing the “champagne glass drop-off transition” and drilling of the fibular head tunnel from anterolateral to posteromedial.
Tibial tunnel preparation	Identification of the “flat spot” on the anterior tibia and the popliteus musculotendinous junction on the posterior tibia and drilling of the tibial tunnel.
Femoral tunnel preparation	Identification of the FCL footprint through traction sutures and creation of the femoral tunnel aiming anteriorly and proximally. Capsulotomy and exposure of the PT footprint on the anterior popliteal sulcus.
Graft fixation sequence	1. Fixation of the thickest extremity of the graft in the femoral tunnel with an interference screw. 2. Passage of the graft into the fibular head tunnel and fixation with an interference screw in neutral rotation, 30° flexion and valgus. 3. Passage of the graft through the loop of the adjustable-length suspension device. 4. Passage of the suspensory system anteriorly through the tibial tunnel and application of the cortical button. 5. Passage of the thin PT limb through the popliteal hiatus and the eyelet of a knotless suture anchor and fixation in its footprint. 6. Tensioning of the PT and PFL at 60° flexion and neutral rotation.

**Abbreviations:** FCL, fibular collateral ligament; PFL, popliteofibular ligament; PT, popliteus tendon.

## Rehabilitation

After PLC reconstruction, patients used a dynamic posterior cruciate ligament hinged knee brace and mobilized non-weight bearing for 6 weeks. Range of motion was initiated postoperatively and gradually progressed to full range of motion. The brace was discontinued at 3 months, and further rehabilitation focused on strength and proprioceptive training. Exercises to prevent posterior sag, as well as external rotation and open-chain exercises, were avoided until this stage. Return to sports was allowed at 9 months, when strength, stability, and knee range of motion were comparable to the contralateral side.

## Final Comments

The original anatomic technique by LaPrade et al. 1 using two separate allografts has proven biomechanical and clinical results. 1 3 5 The Wood et al. 2 modification of this technique, using an adjustable-length suspension device, has the advantage of using a single ST autograft, reducing multiple graft harvesting morbidity. In a recent cadaveric study, both techniques restored normal native varus. Similar outcomes were obtained for external rotation in extension. However, for external rotation at 60° and 90° of flexion, the knee was overtensioned with the Wood et al. 2 technique, meaning special care should be taken during tensioning with this technique. 9

The main limitation of the Wood et al. 2 technique is the length of the harvested ST graft. 2 Indeed, some patients may only reach the necessary length considering the thinnest limb of the ST ( Table 1 ). Our modification, with femoral fixation of the thinnest limb with a knotless suture anchor, allows expansion of this technique to the borderline length graft. An additional benefit is the reduction of the number of tunnels in the femur, decreasing the risk of tunnels coalition. Finally, our sequence, with reconstruction of the FCL first and tensioning of the PT and PFL only after fixation of the PT in its footprint, avoids tendon waste, which may be left over with the sequence described by Wood et al., 2 allowing more incorporation of the graft into the tibial tunnel. The advantages and disadvantages of the authors' modifications are summarized in Table 3 .

**Table 3:** Advantages and disadvantages of the authors' modifications

Advantages	Disadvantages
Knotless suture anchor fixation of the thinnest limb maximizes ST usage allowing anatomic PLC reconstruction with borderline length grafts.	PLC reconstruction with nonanatomic techniques can be performed with shorter ST autografts and are surgically less demanding and invasive.
Knotless suture anchor fixation of the PT excludes the need for a second femoral tunnel reducing the risk of tunnels coalition.	Knotless suture anchor fixation of the PT limb may not be as strong as interference screw fixation. Additional biomechanical studies are needed.
The authors' sequence reduces tendon waste which may be left over with the sequence described by Wood et al maximizing graft usage.	

**Abbreviations:** PLC, posterolateral corner; PT, popliteus tendon; ST, semitendinosus.

In the presence of a graft too short for the technique, biceps femoris tenodesis for reconstruction of the FCL may be considered. 3

Like Wood et al. 2 described, in the setting of combined cruciate ligament reconstruction, we recommend performing the approach to the PLC prior to cruciate ligament reconstruction, to prevent fluid extravasation into the soft tissues and distortion of tissue planes.

The main risks associated with our procedure are the same as the ones described by Wood et al., 2 namely: common peroneal nerve palsy, fibular head fracture, and tunnel coalition with an anterior cruciate ligament femoral tunnel ( Fig. 3 ). To avoid coalition, we recommend following the same tips described in their paper: drilling of the femoral tunnels anteriorly and proximally (30°) as well as second look with the arthroscope after pin insertion to verify no coalition exists before tunnel drilling. 2

## Conclusion

In conclusion, in the presence of a borderline ST for single ST anatomic PLC reconstruction, knotless suture anchor fixation of the thinnest limb, and the sequence described by us should be considered to maximize graft usage.
