# Supplementary material for: Anatomic Posterolateral Corner Reconstruction of the Knee Using Single Semitendinosus Autograft: Technical Note for the Borderline-length Graft
Source: Rev Bras Ortop (Sao Paulo). 2024 Dec 21;59(6):e991–6. doi: 10.1055/s-0044-1793826 (PMC11663074; doi:10.1055/s-0044-1793826)
Supplement: Related texts

# Research article: Reconstrução anatômica do canto posterolateral do joelho com enxerto único de semitendíneo autólogo: Nota técnica para o enxerto de comprimento limítrofe

**Keywords:** joelho, lesões dos tendões, ligamentos articulares

## Abstract

A técnica original de LaPrade para reconstrução anatômica do canto posterolateral do joelho usa dois aloenxertos separados. Mais recentemente, uma modificação dessa técnica, usando um dispositivo de suspensão de comprimento ajustável com um botão cortical para fixação tibial, permite a reconstrução anatômica com um único enxerto autólogo de semitendíneo. Essa modificação é de extrema relevância quando fontes de aloenxerto não estão disponíveis para reconstrução multiligamentar do joelho. Em ambas as técnicas, parafusos de interferência são usados para fixação femoral do ligamento colateral fibular e tendão poplíteo. O comprimento mínimo recomendado para a reconstrução anatômica com semitendíneo único é de 25 cm, mas existem variações anatômicas na população e um semitendíneo mais longo pode ser necessário. De fato, alguns pacientes somente podem atingir o comprimento necessário considerando o membro mais fino do semitendíneo. Nesses pacientes, a fixação femoral do membro mais fino com uma âncora de sutura sem nós, como descrevemos, para o membro do tendão poplíteo, permite a expansão dessa técnica para enxertos autólogos de semitendíneo limítrofes, ao mesmo tempo em que reduz o risco de coalizão de túnel. Também descrevemos uma sequência diferente de etapas: fixação do ligamento colateral fibular no túnel femoral seguido por seu tensionamento e fixação no túnel da cabeça fibular, fixação do tendão poplíteo em sua pegada femoral com uma âncora de sutura sem nós e, finalmente, tensionamento do ligamento poplíteo-fibular e do tendão poplíteo. Essa sequência diferente também ajuda a evitar desperdício de tendão, que pode sobrar, permitindo mais incorporação do enxerto no túnel tibial.

## Introdução

O canto posterolateral (CPL) do joelho consiste em três estabilizadores estáticos principais que restringem o varo e a rotação externa: o ligamento colateral fibular (LCF), o tendão poplíteo (TP) e o ligamento poplíteo-fibular (LPF). O mecanismo de lesão envolve estresse em varo, hiperextensão e torção do joelho. A insuficiência do CPL pode resultar em lesões meniscais e osteoartrite acelerada do compartimento medial. 1 Lesões do CPL raramente são isoladas, sendo tipicamente associadas a lesões em um ou ambos os ligamentos cruzados. No contexto de uma lesão multiligamentar, o não tratamento da insuficiência concomitante do CPL aumenta as forças nos ligamentos cruzados reconstruídos e pode levar à falha cirúrgica. 2 3

Muitas técnicas, tanto anatômicas quanto não anatômicas, foram descritas. Embora alguns estudos apresentem resultados clínicos semelhantes de técnicas anatômicas e não anatômicas, 4 alguns estudos biomecânicos favorecem a reconstrução anatômica do CPL. 1 3 5 A técnica anatômica original, publicada por LaPrade et al., 1 requer dois aloenxertos separados do tendão calcâneo: um para o LCF e LPF e outro para o TP. Modificações da técnica, em que dois autoenxertos separados de isquiotibiais são usados, também foram descritas. 3 6 No entanto, como as lesões do CPL são comumente associadas a outras lesões ligamentares, a coleta de dois isquiotibiais para a reconstrução do CPL aumenta a morbidade da coleta do enxerto. Em 2019, Wood et al *.* 2 descreveram a modificação da técnica de LaPrade et al. 1 usando um dispositivo de suspensão de comprimento ajustável com um botão cortical para fixação tibial dos membros LPF e TP, permitindo a reconstrução do CPL com um único enxerto autólogo de semitendíneo (ST). Esta modificação técnica é um “divisor de águas” quando não há fontes de aloenxerto para reconstrução multiligamentar do joelho. O comprimento mínimo do ST recomendado pelos autores é 25 cm. 2 No entanto, existem variações anatômicas na população 7 8 ( Tabela 1 ) e um ST mais longo pode ser necessário. Em nossa experiência, alguns pacientes somente podem atingir o comprimento necessário considerando o ramo mais fino do ST.

**Tabela 1:** Considerações anatômicas para a estimativa do comprimento do enxerto 1 7 8

Estrutura anatômica	Comprimento necessário do enxerto em milímetros (intervalo)
Ligamento fibular colateral	69,6 (62,6–73,5) 7
Tendão poplíteo	54,5 (50,5–61,2) 7
Ligamento poplíteo-fibular	14,7 (12,2–17,2) 8
Túnel da cabeça fibular	36,3 (31,2–40,7) 7
Túneis femorais	50 1 2
Túnel tibial	Comprimento restante do enxerto

O objetivo desta nota técnica é descrever uma modificação da técnica de Wood et al *.* 2 com fixação femoral do membro mais fino do enxerto com âncora de sutura sem nós ( Fig. 1 ). Também propomos uma sequência diferente de etapas para ajudar a reduzir o desperdício de tendão e permitir maior incorporação do enxerto no túnel tibial.

**Fig. 1:** Joelho esquerdo, representação esquemática da técnica cirúrgica. Vista lateral **(A** ) e vista posterior ( **B** ). **Abreviaturas:** LCF, ligamento colateral fibular; LPF, ligamento poplíteo-fibular; TP, tendão poplíteo.

## Técnica Cirúrgica

O último autor foi o cirurgião principal e desenvolveu a técnica.

O paciente foi posicionado em decúbito dorsal com um apoio lateral à coxa e um apoio para os pés em flexão de 90°. Um torniquete foi colocado ao redor da coxa proximal e inflado conforme necessário.

Como descrito por LaPrade et al. 1 e Wood et al., 2 foi realizada uma abordagem lateral incluindo neurólise do nervo fibular comum. Em seguida, foi feita uma incisão horizontal sobre o tendão da cabeça longa do bíceps femoral com exposição da cabeça fibular e identificação do remanescente do LCF, que foi marcado com suturas de tração, permitindo a identificação de suas pegadas femorais e fibulares. A dissecção subperióstea do aspecto lateral da cabeça fibular foi, então, realizada com exposição da “transição *drop-off* em taça de champanhe” e o túnel da cabeça fibular foi criado (6 × 35 mm), de anterolateral para posteromedial, com o auxílio de um dispositivo adequado tendo como alvo a cabeça fibular (Arthrex, Inc., Naples, FL, EUA). Em seguida, distal e medialmente ao tubérculo de Gerdy, o “ponto plano” na tíbia anterior foi identificado, bem como a junção musculotendínea do poplíteo na tíbia posterior, proximal e medialmente ao túnel da cabeça fibular. Um dispositivo adequado tendo como alvo a tíbia (Arthrex, Inc.) foi usado, e um túnel tibial completo do LPF ao TP foi criado. Uma segunda incisão horizontal foi feita na banda iliotibial, à altura do epicôndilo lateral, com identificação da pegada femoral do LCF, e uma capsulotomia em forma de L permitiu a visualização da pegada do TP no sulco poplíteo anterior. 1 2

O ST coletado, medindo 27 cm, considerando sua extremidade mais fina de 2,5 cm, foi tubularizado com suturas simples separadas para fácil passagem pelos túneis do fêmur, cabeça da fíbula e tíbia. O enxerto foi passado pelo túnel da cabeça da fíbula ( Fig. 2A ) e foi feita uma estimativa inicial do comprimento necessário ( Fig. 2B ). Para fixação femoral do LCF, após o preparo da pegada do LCF ( Fig. 3A ) e colocação do pino guia apropriado ( Fig. 3B ), um túnel de 6 × 25 mm foi criado e uma sutura de passagem foi mantida. Então, procedeu-se à fixação sequencial do enxerto. Primeiro, a extremidade mais espessa do enxerto foi puxada para dentro do túnel femoral e fixada com um parafuso de interferência de 7 × 25 mm (BioComposite FastThread - Arthrex Inc, Fig. 4A ). Em seguida, o enxerto foi fixado na cabeça da fíbula com um parafuso de 7 × 25 mm (BioCompsite FastThread - Arthrex Inc, Fig. 4B ) em rotação neutra, flexão de 30° e estresse em valgo. O enxerto foi então passado pela alça de um dispositivo de suspensão de comprimento ajustável (Attachable Button System - Arthrex Inc). O dispositivo de suspensão foi passado anteriormente pelo túnel tibial e um botão cortical foi aplicado. O membro do TP foi passado ao longo do hiato poplíteo até sua pegada. Como essa extremidade do enxerto não era espessa o suficiente para a fixação do parafuso de interferência, uma âncora de sutura sem nós foi usada. Mais detalhadamente, o membro do TP foi suturado e passado pelo ilhós de uma âncora de sutura sem nós de 4,75 × 19,1 mm, que foi aplicada em sua pegada (BioComposite SwiveLock - Arthrex Inc, Figs. 5A,B ). O TP e o LPF foram então tensionados em flexão de 60° e rotação neutra ( Figs. 6A,B ). Após a fixação de todas as estruturas, a estabilidade do joelho e a amplitude de movimento foram testadas. Após irrigação abundante dos tecidos, a cápsula lateral, a banda iliotibial, o tecido subcutâneo e a pele foram fechados da maneira usual. As principais etapas cirúrgicas são resumidas na Tabela 2 .

**Fig. 2:** Joelho esquerdo, vista lateral. Passagem do enxerto no túnel da cabeça da fíbula ( **A** ) e estimativa inicial do comprimento do semitendíneo necessário para reconstrução com enxerto único ( **B** ). **Abreviaturas:** LCF, ligamento colateral fibular; LPF, ligamento poplíteo-fibular; NPC, nervo peroneal comum; TP, tendão poplíteo.

**Fig. 3:** Preparação da pegada do LCF ( **A** ) e colocação do pino guia antes da perfuração do túnel ( **B** ). **Abreviaturas:** LCA, ligamento cruzado anterior; LCF, Ligamento colateral fibular.

**Fig. 4:** Joelho esquerdo, vista lateral. Fixação do LCF no túnel femoral ( **A** ) com parafuso de interferência de 7 × 25 mm (BioComposite FastThread, Arthrex Inc.). Fixação do LCF no túnel da cabeça da fíbula ( **B** ) com parafuso de 7 × 25 mm (BioComposite FastThread, Arthrex Inc.) em rotação neutra, flexão de 30° e estresse em valgo. **Abreviatura:** LCF, ligamento colateral fibular.

**Fig. 5:** Joelho esquerdo, vista lateral. Fixação do TP em sua pegada femoral ( **A** ) com uma âncora de sutura sem nós de 4,75 × 19,1 mm (BioComposite SwiveLock, Arthrex Inc.). Detalhe da âncora de sutura sem nós com interposição de tendão ( **B** ). **Abreviaturas:** LCF, Ligamento colateral fibular; TP, tendão poplíteo.

**Fig. 6:** Joelho esquerdo, vista lateral. TP e LPF antes do tensionamento ( **A** ) e após o tensionamento ( **B** ). O tensionamento é feito em flexão de 60° e rotação neutra com um botão cortical montado em um dispositivo de suspensão de comprimento ajustável (Attachable Button System, Arthrex Inc.). **Abreviaturas:** LCF, Ligamento colateral fibular; LPF, ligamento poplíteo-fibular; TP, tendão poplíteo.

**Tabela 2:** Resumo das etapas cirúrgicas

Etapa cirúrgica	Detalhes
Posicionamento	Supino com apoio lateral para coxa e apoio para os pés em flexão de 90°.
Abordagem	Abordagem lateral, como descrita por LaPrade et al *.* 1 e Wood et al *.,* 2 incluindo neurólise do nervo peroneal comum.
Preparo do túnel da cabeça fibular	Dissecção subperióstea do aspecto lateral da cabeça da fíbula expondo a “transição *drop-off* em taça de champanhe” e perfuração do túnel da cabeça da fíbula de anterolateral para posteromedial.
Preparo do túnel tibial	Identificação do “ponto plano” na tíbia anterior e da junção musculotendínea poplítea na tíbia posterior e perfuração do túnel tibial.
Preparo do túnel femoral	Identificação da pegada do LCF através de suturas de tração e criação do túnel femoral em direção anterior e proximal. Capsulotomia e exposição da pegada do TP no sulco poplíteo anterior.
Sequência de fixação do enxerto	1. Fixação da extremidade mais espessa do enxerto no túnel femoral com parafuso de interferência. 2. Passagem do enxerto no túnel da cabeça da fíbula e fixação com parafuso de interferência em rotação neutra, flexão de 30° e valgo. 3. Passagem do enxerto pela alça do dispositivo de suspensão de comprimento ajustável. 4. Passagem do sistema suspensor anteriormente pelo túnel tibial e aplicação do botão cortical. 5. Passagem do membro TP fino pelo hiato poplíteo e do ilhós de uma âncora de sutura sem nós e fixação em sua pegada. 6. Tensionamento do TP e LPF em flexão de 60° e rotação neutra.

**Abreviaturas:** LCF, ligamento colateral fibular; LPF, ligamento poplíteo-fibular; TP, tendão poplíteo.

## Reabilitação

Após a reconstrução do CLP, os pacientes usaram uma joelheira articulada dinâmica do ligamento cruzado posterior e se movimentaram sem suporte de peso por 6 semanas. A amplitude de movimento começou no pós-operatório e progrediu gradualmente para a amplitude total de movimento. O uso da órtese foi interrompido aos 3 meses e a reabilitação posterior se concentrou na força e no treinamento proprioceptivo. Exercícios para prevenir a flacidez posterior, além de exercícios de rotação externa e de cadeia aberta, foram evitados até esse estágio. O retorno aos esportes foi permitido aos 9 meses, quando a força, a estabilidade e a amplitude de movimento do joelho eram comparáveis ao lado contralateral.

## Comentários Finais

A técnica anatômica original de LaPrade et al., 1 usando dois aloenxertos separados, teve resultados biomecânicos e clínicos. 1 3 5 A modificação de Wood et al. 2 desta técnica, usando um dispositivo de suspensão de comprimento ajustável, tem a vantagem de usar um único enxerto autólogo de ST, reduzindo a morbidade da coleta múltipla de enxertos. Em um estudo cadavérico recente, ambas as técnicas restauraram o varo nativo normal. Resultados semelhantes foram obtidos para rotação externa em extensão. No entanto, para rotação externa em 60° e 90° de flexão, o joelho foi tensionado em excesso com a técnica de Wood et al., 2 o que significa que cuidados especiais devem ser tomados durante o tensionamento com este procedimento. 9

A principal limitação da técnica de Wood et al. 2 é o comprimento do enxerto de ST coletado. 2 De fato, alguns pacientes somente podem atingir o comprimento necessário considerando o ramo mais fino do ST ( Tabela 1 ). Nossa modificação, com fixação femoral do membro mais fino com uma âncora de sutura sem nós, permite a expansão desta técnica para o enxerto de comprimento limítrofe. Um benefício adicional é a redução do número de túneis no fêmur, diminuindo o risco de coalizão de túneis. Finalmente, nossa sequência, com reconstrução do LCF primeiro e tensionamento do TP e LPF somente após a fixação do TP em sua pegada, evita desperdício de tendão, que pode sobrar com a sequência descrita por Wood et al., 2 permitindo maior incorporação do enxerto no túnel tibial. As vantagens e desvantagens das modificações dos autores estão resumidas na Tabela 3 .

**Tabela 3:** Vantagens e desvantagens das modificações dos autores

Vantagens	Desvantagens
A fixação com âncora de sutura sem nós do membro mais fino maximiza o uso do ST, permitindo a reconstrução anatômica do CPL com enxertos de comprimento limítrofe.	A reconstrução do CPL com técnicas não anatômicas pode ser realizada com enxertos ST autólogos mais curtos e são cirurgicamente menos exigentes e invasivas.
A fixação do TP com âncora de sutura sem nós exclui a necessidade de um segundo túnel femoral, reduzindo o risco de coalizão dos túneis.	A fixação com âncora de sutura sem nós do membro TP pode não ser tão forte quanto a fixação com parafuso de interferência. Há necessidade de mais estudos biomecânicos.
A sequência dos autores reduz o desperdício de tendão que pode sobrar com a sequência descrita por Wood et al., maximizando o uso do enxerto.	

**Abreviaturas:** CPL, canto posterolateral; ST, semitendíneo; TP, tendão poplíteo.

Na presença de enxerto muito curto para a técnica, a tenodese do bíceps femoral para reconstrução do LCF pode ser considerada. 3

Como Wood et al. 2 descreveram, no cenário de reconstrução combinada do ligamento cruzado, recomendamos realizar a abordagem do CPL antes da reconstrução do ligamento cruzado, para evitar extravasamento de fluido para os tecidos moles e distorção dos planos teciduais.

Os principais riscos associados ao nosso procedimento são os mesmos descritos por Wood et al., 2 ou seja, paralisia do nervo fibular comum, fratura da cabeça da fíbula e coalizão do túnel com um túnel femoral do ligamento cruzado anterior ( Fig. 3 ). Para evitar a coalizão, recomendamos seguir as mesmas recomendações descritas no artigo: perfuração dos túneis femorais anterior e proximalmente (30°), bem como uma segunda observação com o artroscópio após a inserção do pino para verificar se não há coalizão antes da perfuração do túnel. 2

## Conclusão

Concluindo, na presença de um ST limítrofe para reconstrução de CPL anatômico de ST único, a fixação com âncora de sutura sem nós do membro mais fino e a sequência descrita por nós devem ser consideradas para maximizar o uso do enxerto.
